# Combining radiation with hyperthermia: a multiscale model informed by *in vitro* experiments

**DOI:** 10.1098/rsif.2017.0681

**Published:** 2018-01-17

**Authors:** S. Brüningk, G. Powathil, P. Ziegenhein, J. Ijaz, I. Rivens, S. Nill, M. Chaplain, U. Oelfke, G. ter Haar

**Affiliations:** 1 Joint Department of Physics at The Institute of Cancer Research and The Royal Marsden NHS Foundation Trust, Sutton, Surrey, UK; 2 Department of Mathematics, College of Science, Swansea University, Swansea,, UK; 3 School of Mathematics and Statistics, University of St Andrews, St Andrews, UK

**Keywords:** hybrid multiscale model, radiotherapy, hyperthermia, cell cycle, cancer, tumour

## Abstract

Combined radiotherapy and hyperthermia offer great potential for the successful treatment of radio-resistant tumours through thermo-radiosensitization. Tumour response heterogeneity, due to intrinsic, or micro-environmentally induced factors, may greatly influence treatment outcome, but is difficult to account for using traditional treatment planning approaches. Systems oncology simulation, using mathematical models designed to predict tumour growth and treatment response, provides a powerful tool for analysis and optimization of combined treatments. We present a framework that simulates such combination treatments on a cellular level. This multiscale hybrid cellular automaton simulates large cell populations (up to 10^7^ cells) *in vitro*, while allowing individual cell-cycle progression, and treatment response by modelling radiation-induced mitotic cell death, and immediate cell kill in response to heating. Based on a calibration using a number of experimental growth, cell cycle and survival datasets for HCT116 cells, model predictions agreed well (*R*^2^ > 0.95) with experimental data within the range of (thermal and radiation) doses tested (0–40 CEM43, 0–5 Gy). The proposed framework offers flexibility for modelling multimodality treatment combinations in different scenarios. It may therefore provide an important step towards the modelling of personalized therapies using a virtual patient tumour.

## 1. Introduction

Cancer is a complex disease, with a variety of approaches available for its treatment. Treatment modalities are often combined to maximize response and to overcome the limitations of individual modalities when used alone. One such example is the combination of radiotherapy (RT) with hyperthermia (HT), i.e. non-ablative sustained heating (41–50°C applied for times up to ≈ 1 h) for the treatment of radiation-resistant tumours, or tumour sub-regions. Heat has a radio-sensitizing effect on cell lines of both normal and malignant origin [1–3]. Heating applied locally to a tumour may therefore enhance treatment outcome without increasing the risk of normal tissue complications. The intracellular mechanisms involved in thermo-radiosensitization are still subject to investigation. However, it is believed that a major cause of this synergism is an inhibition of DNA repair mechanisms by heat, leaving them more vulnerable to radiation-induced DNA strand breaks [4–6]. Radiation induced cell death is a highly regulated cellular process. Depending on factors such as the severity of the damage, cell-cycle stage and cell type, a response cascade, which will not only drive pro-survival repair pathways, but also trigger programmed cell death, is activated [4,7–9]. Once DNA damage is recognized, cell-cycle checkpoint inhibition will prevent cycle progression to allow time for damage repair. Depending on the cell's current stage in its cycle, the allowed duration of cell-cycle arrest, as well as the availability of different DNA repair pathways, varies, leading to differences in repair capacity between cell-cycle stages. However, prolonged activation of repair and cell-cycle arrest drive prodeath pathways, resulting, if repair is unsuccessful, in cell kill via apoptosis, necroptosis or autophagy depending on both the cell type and the severity of the damage [4,7]. If the cell-cycle checkpoint functionality is compromised, unrepaired DNA damage will be inherited by daughter cells and may result in the formation of highly aneuploid cells with abnormal phenotype (giant cells) that eventually die or are unable to reproduce (mitotic catastrophe). This mitotic cell death is therefore not instantaneous but may take several cycles to manifest itself. Cellular senescence, i.e. irreversible cell-cycle arrest, is another potential result of severe DNA damage. Senescent cells do not proliferate but remain metabolically active [4,7]. In order to predict the dynamic treatment response of a tumour, or of a cell population, in general, it is therefore important to consider these various reaction pathways.

Systems oncology simulations [10–14] provide a powerful tool for analysis and optimization of treatment combinations, and make it possible to take inhomogeneities in the delivered heating profiles into account. In general, two types of simulation approaches, continuum and discrete models, are considered. Continuum-based approaches describe macroscopic cell densities and substrate concentrations according to reaction–diffusion processes using sets of partial and ordinary differential equations (PDEs and ODEs) [11]. Discrete approaches, such as cellular automaton models, track individual cells or even sub-cellular elements [11]. These are usually represented as lattice points on a simulation grid with all actions being governed by a set of predefined rules. Hybrid models combine both approaches and model biophysical processes on scales ranging from cellular to macroscopic using a mix of predefined transition rules and PDE-driven processes, such as the diffusion of nutrients or messaging molecules. Recent publications in the field of computational modelling of cancer therapies span a broad range, from modelling cell response *in vitro* [15–17] to modelling angiogenesis and tumour vasculature effects [18,19], the prediction of treatment outcome for patients [20,21] treated with a variety of approaches, and even to describing the evolution of different cancer types [22].

Although several studies have looked into modelling radio- and chemo-therapy response [10,18,23], studies reporting the effects of combination treatments of radiation and heat are few. Several groups have investigated the mathematical modelling of therapy outcome in terms of cell surviving fractions [3,24–26].

We here present an implementation of a hybrid cellular automaton model which simulates the response of cells to heat, RT or combinations of the two, on several different spatio-temporal scales. Temporally, the simulation covers modelling a cell's cycle progression (minutes), cellular division and treatment response (hours), up to the modelling of the growth of the whole population over the course of a treatment (days). Spatially, the simulation ranges from simulating individual cells (μm) to dealing with macroscopic cell culture dishes ( ≈ 10^7^ cells, cm scale). The multiscale nature of the model therefore requires analysis of the effects of single and combination treatments on individual cells, and on the cell population as a whole. The aim of this model was the prediction of response to the treatment of a large-cell population *in vitro*, i.e. to predict the number and distribution of viable cells over time, in order to provide an important first step towards more complex modelling of tumours *in vivo*. This requires finding a compromise between the simplest model that summarizes a cascade of complex biological processes, and a model that is sufficiently complex to describe the observed biological behaviour and is based on known key response pathways.

## 2. Methods

### 2.1. Model implementation

The model presented here is a significant development of the previous model of Powathil *et al.* [23,27], with new implementation in C++. This is a cellular automaton model for the simulation of response to therapy using the recently developed AlphaR survival model designed specifically for calculating cell surviving fractions after multimodality treatments [26]. Besides enabling the introduction of heat as a second treatment modality, the simulation framework has been extended to include dynamic modelling of mitotic cell kill after irradiation. Optimization of the implementation has further allowed an extension of the simulation to large cell populations (of the order of several million cells). This is required for direct comparison between experimental and simulated data. We show that our model can predict the dynamic growth of a treated cell population once key model parameters have been adjusted using experimentally derived *in vitro* data.

#### 2.1.1. Growth modelling

Digital cells are represented as voxels on a two- or three-dimensional lattice depending on the experimental set-up to be simulated. Thus, the diameter of a cell corresponds to the edge length of a voxel. The following discussion of *in vitro* experiments is restricted to the representation of cell monolayers in culture dishes, which are simulated as flat, two-dimensional lattices.

In agreement with the known cell-cycle progression of real cells [28,29], each virtual cell follows the well-known four-stage cycle through *G*_1_, *S*, *G*_2_ and M-phases. Cycle stages are assigned according to an individual cell-cycle timer that is incremented in each time frame according to the predefined growth rate of that cell type. To account for variation in cycle duration for cells within a population, growth rates are assigned using a normal distribution, with a mean growth rate corresponding to the experimentally determined rate for the specific cell type, and a standard deviation of 5% of this value. Upon division, a cell's neighbourhood (i.e. Von Neumann (directly adjacent voxels), or Moore (directly and diagonally adjacent voxels) neighbourhoods) is scanned for free spaces up to third-order neighbours. Moore and Von Neumann neighbourhoods are applied alternately to give circular growth of the cell colony. From all free spaces, an empty voxel is randomly selected for the new position of the daughter cell, with positions closest to the parental cell being occupied first. For simplicity, during these relatively short-term studies (up to two weeks), all cells are assumed to have an infinite life span and unlimited division potential in the absence of external damage, resulting in exponential population growth. However, experimental cellular growth curves *in vitro* (i.e. number of cells present as a function of time) are characterized by an initial lag period during which the cells attach and adapt to their new environment, followed by exponential growth. A lag phase of 2 h was therefore introduced into our simulations. During this phase, digital cells do not progress through their cycle, but may die if treatment is delivered during this time.

**Figure 1.**
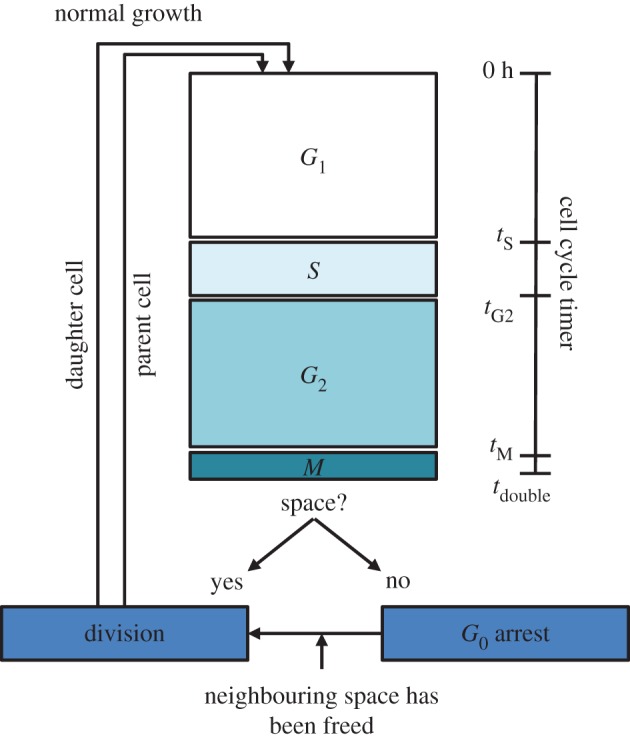
Flow chart showing the implementation of normal cell growth. According to its cycle timer a virtual cell follows a four-stage cycle consisting of *G*_1_, *S*, *G*_2_ and *M*-phases. Once the cycle timer exceeds the threshold time *t*_stage_, the cell is assigned to a new cycle stage. Once the cycle timer reaches the doubling time *t*_double_, the cell will either divide into two cells in stage *G*_1_ or enter the reversible quiescent stage *G*_0_, if no free neighbouring spaces are available. (Online version in colour.)

In a culture dish, a cell population eventually reaches confluence, and proliferation decreases due to a lack of space and increased competition for nutrients. This results in a plateau in the growth curve. A fifth stage, *G*_0_, is introduced to account for this behaviour. *G*_0_ represents a reversible resting stage (quiescence). It is entered if a cell is no longer able to divide due to a lack of free space nearby. Quiescent cells arrest their cycle until neighbouring spaces are vacated, or the cell is killed. For a simulated culture dish of a predefined geometry, the number of cells at which the plateau is reached depends only on the number of voxels present, thus making it necessary to adapt the voxel size to model the shape of a simulated plateauing growth curve correctly. This will be described in detail in §3.1.1. Figure 1 shows a flow chart which describes the simulation of normal cell growth.

### 2.2. Treatment response modelling

#### 2.2.1. Radiotherapy

Radiation is simulated as being delivered homogeneously to all cells in each fraction. Cell survival after radiation, *S*_RT_, is estimated as a function of dose *d* using the AlphaR model [26], extended by a cycle stage-dependent weighting factor *γ* to account for differences in radiation sensitivity at each stage [23].2.1
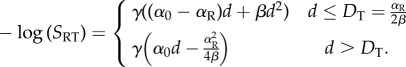
The AlphaR model uses three cell line and treatment-dependent parameters: *α*_0_, *α*_R_ and *β*. These describe a combination of cellular damage (*α*_0_), damage repair (*α*_R_) and reduction of the repair capacity with increasing dose (*β*). *D*_T_ represents a threshold dose above which no damage repair is possible. Whereas for doses lower than *D*_T_, survival is described by a linear-quadratic (LQ) exponential function, the cell survival curve is described by a single exponential for doses exceeding this threshold.

The advantage of the AlphaR model over others, such as the LQ model [30], is its applicability to multimodality therapies. In particular, HT cell survival curves, which are characterized by a strong shoulder followed by an exponentially linear decay, are well described by this model. More information on the cell survival model used is provided in [26].

As for most cell survival models, the AlphaR model relates to survival as measured by clonogenic assays [31]. This assay is conducted several days post treatment, and therefore does not give information about the dynamics of cell damage and repair. In the range of doses used therapeutically, radiation-induced cell killing is not instantaneous, but occurs as a consequence of the cell's inability to undergo division successfully [4,7,8]. This means that irradiated cells may continue to proliferate, become senescent or form giant cells with multiple nuclei (mitotic catastrophe) before undergoing apoptosis. It is important to take this into account as growth restrictions due to space or nutrient limitations, as well as processes such as re-oxygenation (where three-dimensional growth is considered) will be affected by these dying cells.

**Figure 2.**
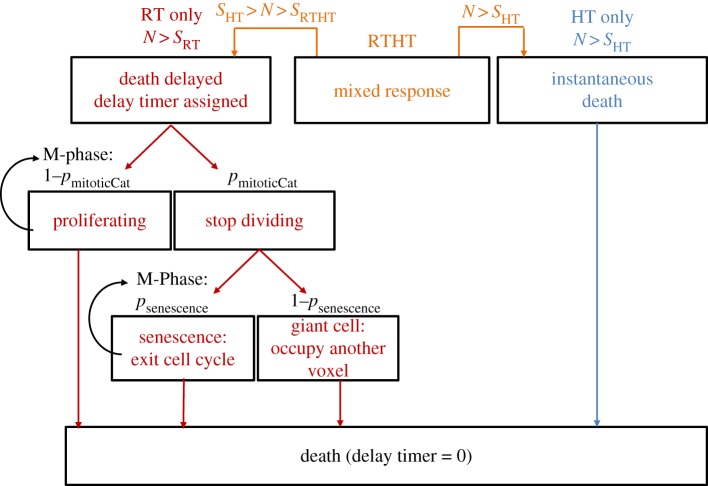
Decision tree used to simulate treatment response to radiation, heat and combination treatments for each individual cell. According to a random number *N*[0, 1] drawn for each cell, a decision is made whether the cell lives (*N* ≤ *S*) or dies. The respective surviving fractions after heat alone, *S*_HT_, radiation alone, *S*_RT_, or combination treatment, *S*_RTHT_, were calculated using equations (2.1), (2.3) and (2.4). Whereas heated cells (HT) are assumed to die instantaneously, irradiated cells (RT) are assigned a time delay before death which is sampled from an exponential distribution. Until this time point is reached, dying cells either keep dividing (with probability 1 − *p*_mitoticCat_), or undergo mitotic catastrophe and become either a giant cell (with probability 1 − *p*_senescence_) or a senescent cell (with probability *p*_senescence_). For combination treatments (RTHT), cells die instantaneously if *N* > *S*_HT_, or follow radiation-induced delayed cell death if *S*_HT_ > *N* > *S*_RTHT_. (Online version in colour.)

Previous simulations have accounted for this observation by artificially increasing the surviving fraction predicted by the cell survival model (e.g. [23,27,32]). However, such an implementation does not reflect actual cellular behaviour, and has not been verified experimentally. In our model, we approximate the dynamics of radiation-induced cell killing using a series of random events as outlined in the decision tree shown in figure 2. Each cell that receives radiation in the simulation, will die with a probability 1-*S*_RT_ (*S*_RT_ being the calculated surviving fraction). However, radiation-induced cell kill is not simulated as being instantaneous, rather, the dying cells are assigned a randomly selected delay period between irradiation and time of death. These delays to cell death are sampled from an exponential distribution with exponent *k*_delay_ which has to be determined from experimental data (see §2.4). During the delay period, cells keep proliferating, but any daughter cells created will die at the same time as their parent. Besides normal proliferation, dying cells can also enter mitotic catastrophe at the end of M-phase, with a probability *p*_mitoticCat_. Cells undergoing mitotic cell death either form giant cells with their size increasing at each attempted division, or they become senescent with probability *p*_senescence_, i.e. they cease to proliferate but are still viable. This allows for a small population of surviving, but non-proliferating cells. It should be stressed that, although motivated by experimental observations, this simulation provides a mathematical construct that provided the best balance between a biologically motivated description and simplifying approximations to build a time efficient simulation. The actual underlying biological processes may, however, be far more complex, and it was the aim of this study to provide a deliberately simple implementation with few model parameters to vary.

#### 2.2.2. Cellular heat response

For the experimental procedure described below, heat treatments are simulated as temporally homogeneous exposures, such as are performed in a thermal cycler. The biological effect of these treatments depends on treatment temperature and duration. To compare different heating profiles, and to quantify the effects of the applied heat distribution at a cellular level, the ‘thermal dose’ concept [33] is used. Thermal dose is defined in terms of the equivalent time at a constant temperature of 43°C (measured in units described as CEM43) required to yield the same number of surviving cells as from a different arbitrary combination of heating time and temperature. It is calculated using a two-case model distinguishing between heating above and below a threshold temperature of 43°C to account for the activation of different biological processes at these temperature levels: for a number of heating steps *i*, heating times *t*_*i*_ at a temperature *T*_*i*_ are expressed in terms of equivalent heating time at 43°C, *t*_43_.2.2
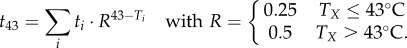
For the calculation of the total thermal dose from a treatment, time steps *t*_*i*_ with temperatures exceeding 40°C are taken into account. In a similar manner to the implementation of the cellular response to radiation, the AlphaR model surviving fraction is used to evaluate the fate of an HT as a function of thermal dose, *S*_HT_(*t*_43_). For this, the dose parameter *d* in equation (2.1) is replaced by the total thermal dose, *t*_43_, according to equation (2.2).2.3
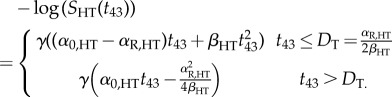
The model parameters *α*_0_, *α*_R_ and *β* are replaced by the cell line-specific parameters determined from HT cell survival curves, *α*_0,HT_, *α*_R, HT_ and *β*_HT_. In the simulation, HT-induced cell killing is modelled to occur instantaneously, and no further cell-cycle delay is applied.

#### 2.2.3. Combination treatments

Similar to the implementation of heat alone, and radiation alone induced cell killing, cell surviving fractions resulting from combined RT and HT treatments, *S*_RTHT_(*d*, *t*_43_), with thermal dose *t*_43_, and radiation dose *d*, are first calculated. As reference data were only available for the LQ branch of the AlphaR model in this case, surviving fractions are calculated as follows:2.4
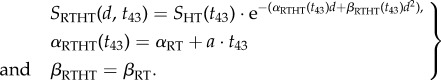
Here, *S*_HT_(*t*_43_) is the surviving fraction due solely to the heat treatment. Heat-induced radio-sensitization is described by a thermal dose-dependent parameter *α*_RTHT_ that increases linearly with thermal dose according to a cell line-dependent slope *a*, whereas *β*_RTHT_ is assumed to be constant. *α*_RT_ (*α*_RT_ = *α*_0,RT_ − *α*_R,RT_), and *β*_RT_ refer to radiation only treatments. These thermal dose dependencies were analysed in detail in [26] for the cell line used here, and in [34–36] using a very similar parametrization for different cell lines.

In simulating the dynamic cell kill in response to combination treatments, cells are immediately ‘killed’ according to the surviving fractions derived from the heat contribution of the treatment, i.e. a fraction of 1 − *S*_HT_ cells is removed from the grid. Of the remaining cells, 1 − *S*_RTHT_(*d*, *t*_43_)/*S*_HT_(*t*_43_) (equation (2.4)) are assigned a time delay before cell death sampled from an exponential distribution as described for the simulation of RT alone cell kill (see flow chart in figure 2).

### 2.3. Experimental procedure for calibration and validation experiments

A number of experiments were performed to calibrate the simulation framework used to model the response of the colorectal carcinoma cell line HCT116. These included growth curves, cell-cycle analysis and clonogenic cell survival assays (published in [26]).

For all experiments, HCT116 cell monolayers were grown at 37°C in McCoy's 5A medium (Gibco, Paisley, UK) supplemented with 10% fetal bovine serum (PAN Biotech, UK) and 1% antibiotics (50 U ml^−1^ each of penicillin, streptomycin B and amphotericin B (Sigma Aldrich, Poole, UK)) in a humidified atmosphere containing 5% CO_2_. Cells were passaged twice weekly using the gentle detaching agent Accutase (Gibco, Paisley, UK). Regular screening for mycoplasma and bacterial contamination was undertaken, and cells in exponential growth phase between passages 10 and 20 were used for experiments.

For treatments, cells were detached, concentrated to give a suspension of 5 × 10^6^ cells ml^−1^ and transferred to sterile, thin-walled PCR tubes (VWR, Lutterworth, UK) in 60 μl volumes. For irradiation, tubes containing cells were embedded in a solid water sample holder and irradiated using a small animal radiation research platform (SARRP, X-Strahl, Camberley, UK) at a dose rate of 63 mGy s^−1^. Heating to 46°C for 5 min was performed in a Biorad Tetrad2 DNA Engine PCR thermal cycler (Hercules, CA, USA) using the techniques described in [26,37]. Cells were kept on ice before, between and after treatments to minimize cellular activity during waiting periods. For combination treatments, cells were first irradiated, and then heated within 20 min of this irradiation. It was confirmed experimentally that if heating was applied within 30 min, the delay between irradiation and heating made no difference to the subsequent clonogenic cell survival [26]. For fractionated treatments, plates seeded with the required number of cells were irradiated every 24 h using an X-ray cabinet (X-Strahl, Camberley, UK, dose rate 63 mGy s^−1^).

#### 2.3.1. Growth curves

Cell growth curves were acquired by seeding triplicates of treated cells in 24- or six-well plates depending on the expected number of surviving cells. Seeding densities ranged from 2 × 10^4^ cells/24-well plate to 5.4 × 10^5^ cells/six-well plate depending on the treatment given. Cell number was determined at various time points (24–200 h) after treatment by counting detached cells in a haemocytometer. A minimum of 100 cells in a minimum of three 1 × 1 mm^2^ were counted for each replicate. For each data point, the average cell count and corresponding standard deviations of three replicates were taken. Growth curves were also acquired at comparable cell densities in 96-well plates. Cells in 96-well plates were fixed with ice-cold 10% trichloroacetic acid, and subsequently stained with sulforhodamine B (Sigma Aldrich, Poole, UK) before being imaged with bright-field microscopy to study cell morphologies.

#### 2.3.2. Cell-cycle analysis

For cell-cycle analysis, cells were detached, washed in ice-cold phosphate-buffered saline and fixed in ice-cold 70% ethanol. Fixed cells were treated with RNase to minimize non-specific background staining, and stained with the DNA-intercalating dye propidium iodine (PI, Sigma Aldrich, Poole, UK) for 15 min at room temperature in the dark prior to analysis using flow cytometry in an LSRII analyser (BD, Franklin Lakes, NJ). DNA content of at least 10^4^ cells was measured in this way and data were collected using DiVa (BD, Franklin Lakes, NJ). The resultant histograms were fitted by the Watson pragmatic model in FlowJo^®^ to obtain cell-cycle distribution proportions.

#### 2.3.3. Clonogenic assays

Clonogenic cell survival data have been published in [26] for HCT116 cells. Table 1 summarizes all parameters used for calculating clonogenic surviving fractions for possible treatment scenarios for this cell line. For RT and RTHT treatments, the LQ arm of the AlphaR model was sufficient to describe the cell survival data obtained for the radiation doses used here. Therefore, no individual values for *α*_0_, and *α*_R_ are needed, and the difference between these parameters, (i.e. *α* = *α*_0_ − *α*_R_), is given. For HT treatments *α*_0_ = *α*_R_ (see [26] for details).

**Table 1.** AlphaR-model parameters (with their time and temperature dependence) used to simulate the treatment response of HCT116 cells.

	*α* = *α*_0_ − *α*_R_	*α*_0_	*β*
RT	0.5 Gy^−1^	—	0.042 Gy^−2^
HT	0	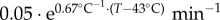	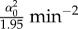
RTHT	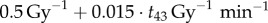	—	0.042 Gy^−2^

### 2.4. Parameter fitting upon model calibration

Where it was not possible to measure model parameters experimentally, these were adjusted to yield the best overall agreement in terms of coefficients of determination *R*^2^ between simulated and reference data during calibration of the model. For model validation, no further changes were made to the parameters used. Table 2 gives an overview of all parameters used in this simulation and the method used for their determination.

**Table 2.** Summary of all parameters used in this simulation framework together with the method used for parameter estimation.

parameter	calibration method
*α*, *α*_0_, *α*_R_,*β*	clonogenic assay
growth rate	growth curve
number of cells in plateau phase	growth curve
cell-cycle distribution	flow cytometry
initial number of cells *N*_0_	haemocytometer count
*p*_mitoticCat_, *p*_senescence_, *k*_delay_	fit of *R*^2^

## 3. Results

### 3.1. Model calibration

#### 3.1.1. Cell-cycle distribution and growth

The simulation framework was first calibrated to model the growth and treatment response of the colorectal carcinoma cell line HCT116. Cellular growth curves were used to determine cell doubling time and voxel size, while flow cytometry was used to obtain information about the cell-cycle distribution.

**Figure 3.**
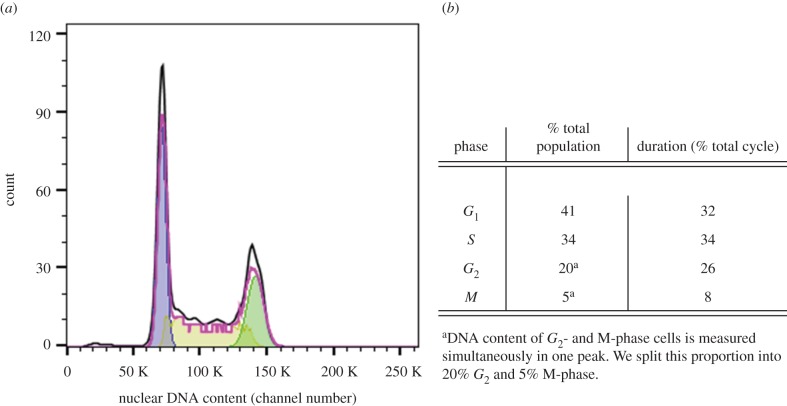
(*a*) Histogram of the cellular DNA content obtained by flow cytometry. From a fit of the experimental data using the Watson pragmatic model, the proportions of singlets corresponding to cells in *G*_1_-phase (first peak, purple), cells in the process of reproducing their DNA in S-phase (middle part, yellow), and doublets corresponding to cells in *G*_2_- or M-phase are determined (second peak, green). The experimental data (black) and the model fit (pink) are shown. (*b*) Table of cell-cycle distribution, and cycle stage duration as obtained by flow cytometry analysis of untreated HCT116 cells. (Online version in colour.)

Figure 3 shows the resulting histogram of the cellular DNA content separated into *G*_1_, *S* and *G*_2_/*M* phase, as well as a table of the percentage of cells in, and duration of, each cycle phase. As flow cytometry cannot distinguish between cells in M- or *G*_2_-phase (because they have the same DNA content), it was assumed that the 25% of cells in both phases is split into 20% *G*_2_− , and 5% M-phase.

**Figure 4.**
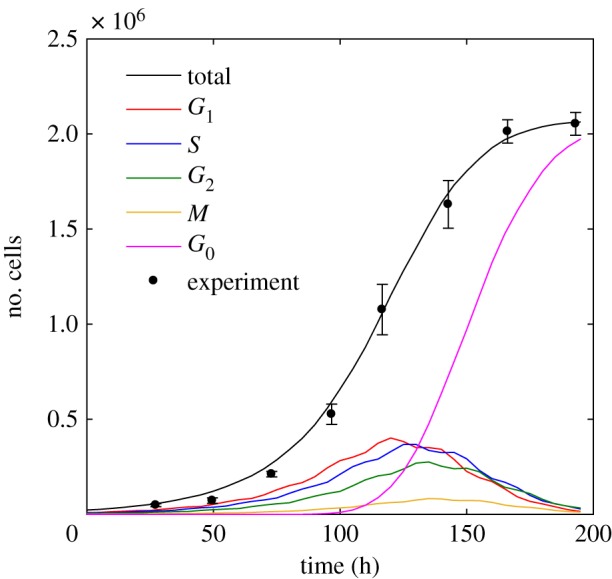
Simulated and experimentally determined growth curves for untreated HCT116 cells in 24-well plates; 2.3 × 10^4^ cells with a doubling time of 19.5 h on a 15.6 mm diameter circular two-dimensional grid (corresponding to a diameter of 1625 voxels) were simulated using the cell-cycle distribution information given in figure 3. The experimental data points (black circles, means and standard deviations), as well as the simulated total cell numbers and simulated contributions from the different cell-cycle stages (solid lines) are shown.

Figure 4 shows the corresponding growth curve for 2.3 × 10^4^ cells seeded in a 24-well plate (15.6 mm well diameter) along with the calibrated simulation. Data points and error bars correspond to mean and standard deviation values from three replicates of a single experiment. An average doubling time of 19.5 ± 1 h was measured for HCT116 cells. A good simulation of the growth curve plateau was obtained for voxel sizes of 9.6 × 9.6 μm^2^ and 12 × 12 μm^2^ if cells in 24-well or six-well plates were simulated. Differences in voxel size determined from the plateau cell density in the two well types may be due to changes in nutrient medium volume-to-surface-ratios, as well as to variations in frequency of medium change for different samples.

#### 3.1.2. Treatment response

**Figure 5.**
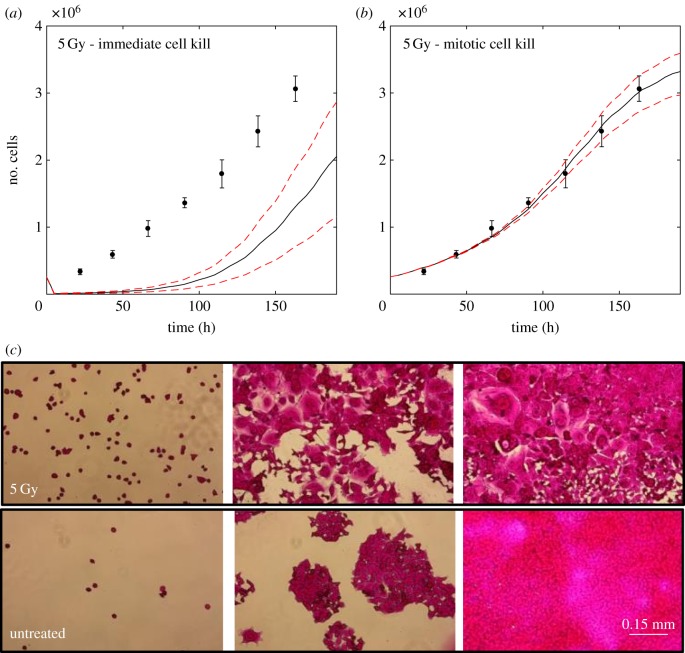
Calibrated simulation of a growth curve of 2.6 × 10^5^ irradiated (5 Gy) HCT116 cells seeded in six-well plates with a diameter of 34.8 mm (corresponding to a grid diameter of 2900 voxels). The experimental data points (black circles) are shown, as well as the simulated total cell numbers (solid lines) together with simulation of the 95% confidence bounds of the surviving fractions used (dashed lines, *S*_5 Gy_ = 0.03 (0.02, 0.04)). (*a*) Simulation assuming instantaneous cell kill greatly underestimated the total number of cells. (*b*) Simulation assuming controlled delayed cell kill via mitotic catastrophe was successfully calibrated to match the experimental data. (*c*) Photographs of fixed irradiated, and untreated cells at three different growth stages (initially seeded, sub-confluent (96 h) and confluent wells (144 h)). The pink stain corresponds to cellular protein stained by sulforhodamine B. A change in morphology from normal phenotype to a mixture of normal and aneuploid cells is seen for irradiated cells.

The simulation is first adapted to match the growth of HCT116 cells irradiated with 5 Gy. This was achieved by adjusting the exponent *k*_delay_, and the probability *p*_mitoticCat_ that characterize the distribution of delay times to cell death, and the probability of undergoing mitotic catastrophe after radiation treatments. Figure 5*a*,*b* shows the resulting growth of 2.6 × 10^5^ irradiated cells seeded in a six-well plate with the corresponding simulation assuming (*a*) instantaneous cell kill or (*b*) controlled delayed cell kill via mitotic catastrophe using *k*_delay_ = 0.009 h^−1^, and *p*_mitoticCat_ = 0.2. Neither *p*_senescence_ nor *γ* significantly changed the simulation result for these conditions. The probability of cellular senescence, *p*_senescence_, was fixed at a value of 5% since we have assumed a small contribution from senescent cells. The sensitivity between different cycle stages was assumed to be a constant ratio of 1.5, meaning that surviving fractions *S*_phase_ differ by powers of 1.5 relative to each other: *S*_S_ = 1.5 · *S*_G1_ = 1.5^2^ · *S*_G2_ = 1.5^3^ · *S*_M_. This corresponds to factors, *γ*, ranging from 0.85 (least sensitive, S-phase) to 1.39 (most sensitive, M-phase).

It is clear that a simulation which assumes instantaneous cell death would greatly underestimate the total number of cells, whereas *in vitro* and *in silico* experiments are in very good agreement within the boundaries of the 95% confidence intervals of the calculated surviving fraction (*S*_5Gy_ = 0.04(0.03,0.05)) when delayed cell kill is assumed. Photographs of fixed, irradiated and untreated cells at three different growth stages (initially seeded, subconfluent (96 h) and confluent wells (144 h)) are shown in figure 5*c*. In the irradiated samples, giant cells are clearly visible at the subconfluent, and confluent stages.

### 3.2. Model validation and application

**Figure 6.**
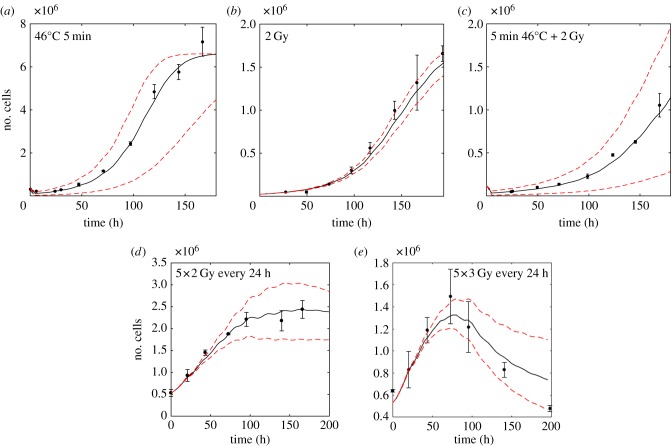
Comparison of experimental and simulated growth curves for HCT116 cells treated with different modalities: (*a*) heating at 46°C for 5 min (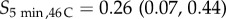, 3 × 10^5^ cells seeded in six-well plates). (*b*) 2 Gy radiation (*S*_2 Gy_ = 0.31 (0.25, 0.37), 2.4 × 10^4^ cells seeded in 24-well plates). (*c*) Combination of 2 Gy radiation and heating for 5 min at 46°C (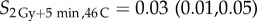), 1.2 × 10^5^ cells seeded in six-well plates). (*d*) Five fractions of 2 Gy radiation given every 24 h (*S*_2 Gy_ = 0.31 (0.25, 0.37)), 5.4 × 10^5^ cells seeded in six-well plates). (*e*) Five 3 Gy fractions of radiation given every 24 h (*S*_3 Gy_ = 0.15 (0.11, 0.19)), 5.4 × 10^5^ cells seeded in six-well plates). Cells were simulated as having a mean doubling time of 19.5 h. The experimental data points (black circles, means and standard deviations) are shown, as well as the simulated total cell numbers (solid lines) and simulation of the 95% confidence bounds of the surviving fractions used (dashed lines). (Online version in colour.)

**Figure 7.**
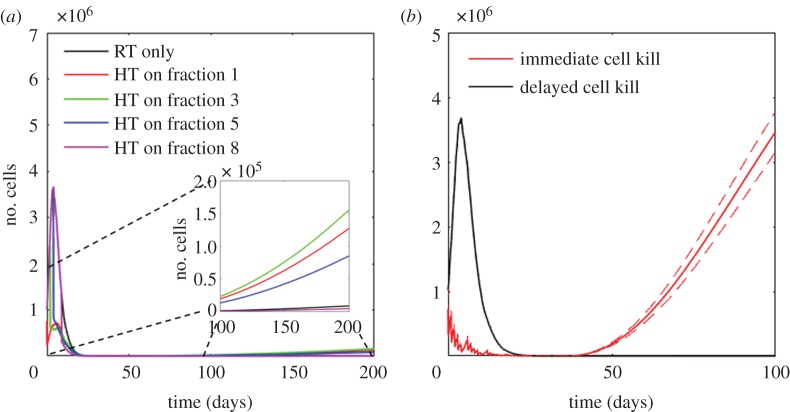
(*a*) Simulation results of fractionated treatments using 30 fractions of 2 Gy, of which one fraction is given in combination with a thermal dose treatment of 40 CEM43 at different days within the overall treatment period as indicated by the legend (5 × 10^6^ cells initially). The means of 500 simulation runs are shown. Inset: expanded view of the population growth 100–200 days post treatment. Although the overall delivered radiation and thermal doses are the same, depending on the combination of the treatment fractions, the biological response may vary. (*b*) Fractionated RT treatment of 30 fractions of 2 Gy simulated using delayed (black lines) or instantaneous (red lines) cell kill (1 × 10^6^ cells initially). Treatment was delivered 5 days a week, Monday to Friday, over a time course of 30 days. The means (solid lines) and standard deviations (dashed lines) of 500 simulation runs are shown. Whereas a simulation using delayed cell kill modelling predicts complete cell killing, a simulation that assumes immediate cell death would predict tumour regrowth.

HCT116 growth curves for heated (5 min at 46°C), irradiated (2 Gy, 5 × 2 Gy, 5 × 3 Gy), and combination treated cells (2 Gy irradiation, and heating for 5 min at 46°C) are simulated and compared to the corresponding experimental growth curves (figure 6). The calculated surviving fractions are shown with 95% confidence intervals: *S*_2 Gy_ = 0.31 (0.25, 0.37), *S*_3 Gy_ = 0.15 (0.10, 0.20), 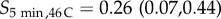, 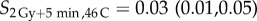. For all treatment scenarios, simulation and experimental data are in good agreement. Although there seems to be a small offset between simulated and measured growth curves for cells treated with 2 Gy in a single fraction, this may be explained by a small deviation of the experimental surviving fraction from the average surviving fraction calculated by the AlphaR model. However, the experimental result lies well within the 95% confidence bounds of the predicted growth curves. In particular, for fractionated treatments, the overall shape of the growth response curve is well described for both 2 and 3 Gy fractions. Having shown that the simulation framework accurately predicts different homogeneous irradiation and heat treatments, it can now be used, for example, to predict the growth response to different fractionation combinations of radiation and heat. Since the simulation uses a stochastic succession of events, the average and standard deviations of 500 runs are taken for each simulated treatment course. Figure 7 shows the influence of choosing a different time point for heat application within the overall treatment schedule involving 30 fractions of 2 Gy radiation in combination with a single combined treatment (thermal dose of 40 CEM43). Depending on the time of adding the heat fraction, the overall treatment success in terms of time until tumour regrowth may be changed slightly due to the alterations in the proportions of quiescent and proliferating cells. Based on this observation, this simulation framework may help to identify the best treatment order for RTHT therapies for different proportions of proliferating and quiescent cells at treatment onset.

We also demonstrate that using instantaneous rather than delayed cell killing in response to RT may change the overall treatment outcome prediction of fractionated RT treatments alone (figure 7*b*) due to different numbers of regrowing cells in the two simulations. If instantaneous cell kill is considered, more space is available to allow cell division compared with the delayed cell kill simulation, where more cells remain quiescent for a longer period of time.

## 4. Discussion

The cellular automaton model presented provides flexibility for application to the study of a number of different situations, both *in vivo* and *in vitro*. We have successfully calibrated the model for single and multimodality therapies of heat and ionizing radiation and could accurately predict treatment response for one particular cell line. This was achieved by introducing a delay before cell death, rather than solely instantaneous death, to describe the cellular response to irradiation. This is an important difference between our, and previous [27], implementations.

Cellular growth curves offer the possibility of testing the simulation in terms of the dynamic development of a treated population over time, rather than providing an absolute surviving fraction only at the fixed time point post treatment provided by clonogenic assays. Since most cell survival models, such as the AlphaR or the LQ, are based on clonogenic cell survival data, they predict the long-term surviving fraction, but cannot give any information on how the population develops towards this endpoint. Growth curves, on the other hand, provide dynamic information, but it can be difficult to extrapolate from them to the overall surviving fraction. Therefore, a combination of surviving fraction, as measured by clonogenic assays, and growth curve data captures a more complete picture. It is, however, difficult to measure cell growth within the first few hours after cell seeding due to the lag between cell seeding, their attachment and exponential growth. Although there might well be differences in the lag time of treated and untreated cells, all experiments have here been allocated a constant lag period of 2 h, resulting in slight differences between experiment and simulation during the initial growth phase. While correct modelling of the initial growth phase may be difficult due to this lag time, the exponential and plateau phase of growth curves are accessible for model calibration. However, the plateau cell density depends strongly on the nutrients provided, i.e. the frequency of medium renewal and its overall volume. When fresh medium is continuously provided, cells in a confluent layer may continue to grow. These new cells may attach on top of other cells. As only a perfect monolayer of virtual cells of rigorously controlled size and shape is considered in this simulation, the voxel size used must be carefully adapted to the properties of the dishes. Although the voxel size, and therefore the maximum number of cells per well may be of importance for simulating fully confluent cells *in vitro*, the effects may be less important for tumours *in vivo*.

Figures 5 and 7*b* show that it is essential to consider the impact of delayed reproductive cell death, because instantaneous cell death greatly underestimates the number of living cells during the first days after treatment. This may influence simulation results, e.g. when studying the effects of tissue reoxygenation and tumour growth response, since immediate removal of all dying cells would allow faster repopulation and enhanced reoxygenation of the tumour compared to delayed cell kill simulations. For long-term studies of single treatment fractions, these delayed effects should be negligible since the surviving population will eventually provide the majority of proliferating cells. The parameters used to describe the dynamics of delayed cell kill (*k*_delay_, *p*_mitoticCat_ and *p*_senescence_) have here been customized to fit the data from HCT116 cells. Although it might be possible that some, or all, of these parameters are similar for other cell types, their accurate modelling would require re-calibration of these parameters.

The calculation of surviving fractions strongly depends on the biological model parameters used, i.e. *α*_0_, *α*_R_ and *β*, in the case of the AlphaR model. These parameters are subject to relatively large uncertainties and individual experiments often deviate from the predicted average surviving fraction. This may be a feature of clonogenic assays, which are very sensitive to experimental error (e.g. from cell counting and pipetting uncertainties), and strongly depend on the experimental conditions, such as incubation time, cell-cycle distribution and density, and cell health in general. This results in relatively large intra- and inter-experimental differences, leading to the large reported confidence bounds of the surviving fractions for HT and combination treatments.

All biological results presented were obtained for a commercially available human cancer cell line. As all reference experiments were performed by us on the same batch of cells, rather than using previously published data from a number of sources, our calibration benefits from a consistent dataset, which is immune to errors arising from differences in experimental techniques between different laboratories. Based on the current results, simulations of other cell types may either be calibrated using the techniques presented here, or a sensitivity analysis of the parameters used could be performed to understand the importance of individual parameters on the overall simulation outcome (see electronic supplementary material, appendix A). This would allow study of the effects of heterogeneous cell populations such as seen in tumours *in vivo*.

Although the model provides good predictions within the calibrated dose range, it should be stressed that doses outside the range of standard therapies, in particular ablative thermal doses and large single fraction RT treatments, have not yet been studied, and may be subject to different cellular response mechanisms which are not captured by this implementation. Also, a number of other parameters that influence the overall cell surviving fraction, e.g. the irradiation dose rate or time interval between HT and RT treatments, are not yet included. A recent publication by van Leeuwen *et al.* [38] touched on this discussion and proposed an exponentially decaying influence of heat-induced radiosensitization as a function of time between HT and RT application. However, there is ongoing discussion concerning the decay rate of heat-induced radiosensitization which is considered to be cell line dependent, and may be greatly influenced by physiological factors such as blood flow leading to different parameter estimations between *in vivo* and *in vitro* applications even for the same tumour type. Van Leeuwen *et al.* recommended using a decay rate of the order of 2 h. Since in our simulation, RT and HT are considered to be given simultaneously, and consecutive radiation fractions are given at least 24 h apart from one another, we believe that our results remain valid, given we have included no time factor in the framework at this stage. Further experimental validation would be required if more complex heating and radiation schedules using shorter time intervals between treatment fractions, or longer time gaps between heat and radiation application, are of interest.

One particularly interesting application of the model is, therefore, the verification of the thermal dose concept for temperatures exceeding 48°C. Owing to the exponential relation of thermal dose on treatment temperature, even small deviations from the proposed mathematical descriptions translate into large differences in thermal dose and cell survival in this temperature range. With uniform heating approaches, it is impossible to verify the thermal dose concept for very high temperatures accurately, since thermal dose uncertainties originating from heating and cooling gradients are difficult to account for in such cases. The proposed framework can be applied to the simulation of different cell distributions in order to calculate an overall probability for the expected cell survival.

Moreover, while the combination of focused ultrasound mediated heating [39,40] with RT provides a promising approach on paper, it is difficult to verify experimentally at a cellular level. For treatment and experiment planning, as well as for determining effective dose and exposure prescriptions, it is essential to first quantify the biological effects at a cellular level. Systems oncology simulations may provide a very useful tool for analysis of the effects of different, inhomogeneous (in space and time) heat and radiation distributions, for which the averaged cell surviving fractions for a subset of carefully selected scenarios can be verified experimentally. The simulation framework presented here was designed specifically for such applications. It is possible to simulate the whole experimental procedure of an *in vitro* focused ultrasound experiment, as well as to analyse the expected overall treatment response of the cell population.

## 5. Conclusion

We have demonstrated a cellular automaton model that is suitable for accurate modelling of the dynamic response to separate, or combined, heat and RT treatments. The inclusion of delayed rather than instantaneous, cell kill after irradiation may impact on simulations which aim to study the effects of reoxygenation and tumour progression, and should therefore be taken into account. We have presented a simple implementation for enabling such modelling, and verified our framework against a consistent experimental dataset thus making this simulation framework a reliable basis for future applications where the effects and optimized treatment protocols for combination treatments *in vivo* are studied.

## Supplementary Material

Sensitivity analysis of the parameters used

## Data accessibility

The code for the framework is part of an ongoing PhD project, which will rely on its further development of the code. Making the code publicly available is an unacceptable risk to the novelty of the students project but we can provide copies to individuals upon request.

## References

[1] Horsman M, Overgaard J 2007 Hyperthermia: a potent enhancer of radiotherapy. Clin. Oncol. 19, 418–426. (10.1016/j.clon.2007.03.015) 17493790

[2] Kampinga HH 2006 Cell biological effects of hyperthermia alone or combined with radiation or drugs: a short introduction to newcomers in the field. Int. J. Hyperthermia 22, 191–196. (10.1080/02656730500532028) 16754338

[3] Dikomey E, Jung H 1991 Thermal radiosensitization in cho cells by prior heating at 41–46°C. Int. J. Radiat. Biol. 59, 815–825. (10.1080/09553009114550711) 1672368

[4] Lauber K, Brix N, Ernst A, Hennel R, Krombach J, Anders H, Belka C 2015 Targeting the heat shock response in combination with radiotherapy: sensitizing cancer cells to irradiation-induced cell death and heating up their immunogenicity. Cancer Lett. 49, 1–21. (10.1016/j.canlet.2015.02.047) 25754814

[5] Kampinga HH, Dikomey E 2001 Hyperthermic radiosensitization: mode of action and clinical relevance. Int. J. Radiat. Biol. 77, 399–408. (10.1080/09553000010024687) 11304434

[6] Roti JLR 2008 Cellular responses to hyperthermia (40–46°C): cell killing and molecular events. Int. J. Hyperthermia 24, 3–15. (10.1080/02656730701769841) 18214765

[7] Roos WP, Thomas AD. Kaina B 2016 DNA damage and the balance between survival and death in cancer biology. Nat. Rev. Cancer 16, 20–33. (10.1038/nrc.2015.2) 26678314

[8] Lauber K, Ernst A, Orth M, Herrmann M, Belka C 2012 Dying cell clearance and its impact on the outcome of tumor radiotherapy. Front. Oncol. 2, 116 (10.3389/fonc.2012.00116) 22973558 PMC3438527

[9] Pawlik TM, Keyomarsi K 2004 Role of cell cycle in mediating sensitivity to radiotherapy. Int. J. Radiat. Oncol. Biol. Phys. 59, 928–42. (10.1016/j.ijrobp.2004.03.005) 15234026

[10] Enderling H, Chaplain MaJ, Hahnfeldt P 2010 Quantitative modeling of tumor dynamics and radiotherapy. Acta Biotheor. 58, 341–53. (10.1007/s10441-010-9111-z) 20658170

[11] Lowengrub JS, Frieboes HB, Jin F, Chuang Yl, Li X, Macklin P, Wise SM, Cristini V 2010 Nonlinear modelling of cancer: bridging the gap between cells and tumours. Nonlinearity 23, R1–R9. (10.1088/0951-7715/23/1/R01) 20808719 PMC2929802

[12] Deisboeck TS, Wang Z, Macklin P, Cristini V 2011 Multiscale cancer modeling. Annu. Rev. Biomed. Eng. 13, 127–155. (10.1146/annurev-bioeng-071910-124729.Multiscale) 21529163 PMC3883359

[13] Hatzikirou H, Chauviere A, Bauer AL, Leier A, Lewis MT, Macklin P, Marquez-Lago TT, Bearer EL, Cristini V 2012 Integrative physiological oncology. Wiley Interdiscip. Rev. Syst. Biol. Med. 4, 1–14. (10.1002/wsbm.158.Integrative) 21853537 PMC3622087

[14] Wang Z, Deisboeck TS 2014 Mathematical modeling in cancer drug discovery. Drug Discov. Today 19, 145–50. (10.1016/j.drudis.2013.06.015) 23831857

[15] Richard M, Kirkby K, Webb R, Kirkby N 2007 A mathematical model of response of cells to radiation. Nucl. Instrum. Meth. B 255, 18–22. (10.1016/j.nimb.2006.11.077)

[16] Ribba B, Colin T, Schnell S 2006 A multiscale mathematical model of cancer, and its use in analyzing irradiation therapies. Theor. Biol. Med. Model. 3, 1–19. (10.1186/1742-4682-3-7) 16472396 PMC1388194

[17] Kam Y, Rejniak KA, Anderson ARA 2012 Cellular modeling of cancer invasion: Integration of *in silico* and *in vitro* approaches. J. Cell. Physio. 227, 431–438. (10.1002/jcp.22766) PMC3687536 21465465

[18] Grogan Ja, Markelc B, Connor AJ, Muschel RJ, Pitt-Francis JM, Maini PK, Byrne HM 2016 Predicting the influence of microvascular structure on tumour response to radiotherapy. IEEE Trans. Biomed. Eng. 64, 504–511. (10.1109/TBME.2016.2606563) 27623567

[19] Perfahl H, Byrne HM, Chen T, Estrella V, Alarcon T, Lapin A, Gatenby RA, Gillies RJ, Lloyd MC, Maini PK, Reuss M, Owen MR 2011 Multiscale modelling of vascular tumour growth in 3D: The roles of domain size and boundary conditions. PLoS ONE 6, e14790 (10.1371/journal.pone.0014790) 21533234 PMC3076378

[20] Enderling H, Chaplain MAJ 2014 Mathematical modeling of tumor growth and treatment. Curr. Pharm. Des. 20, 4934–4940. (10.2174/1381612819666131125150434) 24283955

[21] Rockne RC *et al.* 2015 A patient-specific computational model of hypoxia-modulated radiation resistance in glioblastoma using ^18^F-FMISO-PET. J. R. Soc. Interface 12, 20141174 (10.1098/rsif.2014.1174) 25540239 PMC4305419

[22] Osborne JM 2015 Multiscale model of colorectal cancer using the cellular Potts framework. Cancer Inform. 14, 83–93. (10.4137/CIN.S19332) PMC4598229 26461973

[23] Powathil GG, Swat M, Chaplain MAJ 2015 Systems oncology: towards patient-specific treatment regimes informed by multiscale mathematical modelling. Sem. Cancer Biol. 30, 13–20. (10.1016/j.semcancer.2014.02.003) 24607841

[24] Scheidegger S, Fuchs HU, Zaugg K, Bodis S, Füchslin RM 2013 Using state variables to model the response of tumour cells to radiation and heat: a novel multi-hit-repair approach. Comput. Math. Methods Med. 2013, 587543 (10.1155/2013/587543) 24396395 PMC3876778

[25] Kok HP, Crezee J, Franken NAP, Stalpers LJA, Barendsen GW, Bel A 2014 Quantifying the combined effect of radiation therapy and hyperthermia in terms of equivalent dose distributions. Int. J. Radiat. Oncol. Biol. Phys. 88, 739–745. (10.1016/j.ijrobp.2013.11.212) 24411189

[26] Brüningk SC, Ijaz J, Rivens I, Nill S, Oelfke U, ter Haar G 2017 A comprehensive model for heat-induced radio-sensitisation. Int. J. Hyperthermia 1–11. (10.1080/02656736.2017.1341059) PMC5989161 28641499

[27] Powathil GG, Adamson DJa, Chaplain MaJ 2013 Towards predicting the response of a solid tumour to chemotherapy and radiotherapy treatments: clinical insights from a computational model. PLoS Comput. Biol. 9, e1003120 (10.1371/journal.pcbi.1003120) 23874170 PMC3708873

[28] Vermeulen K, Van Bockstaele DR, Berneman ZN 2003 The cell cycle: a review of regulation, deregulation and therapeutic targets in cancer. Cell Prolif. 36, 131–149. (10.1046/j.1365-2184.2003.00266.x) 12814430 PMC6496723

[29] Branzei D, Foiani M 2008 Regulation of DNA repair throughout the cell cycle. Nat. Rev. Mol. Cell Biol. 9, 297–308. (10.1038/nrm2351) 18285803

[30] Fowler JF 1989 The linear-quadratic formula and progress in fractionated radiotherapy. Br. J. Radiol. 62, 679–694. (10.1259/0007-1285-62-740-679) 2670032

[31] Franken NAP, Rodermond HM, Stap J, Haveman J, van Bree C 2006 Clonogenic assay of cells *in vitro*. Nat. Protoc. 1, 2315–2319. (10.1038/nprot.2006.339) 17406473

[32] Enderling H, Anderson ARa, Chaplain MaJ, Munro AJ, Vaidya JS 2006 Mathematical modelling of radiotherapy strategies for early breast cancer. J. Theor. Biol. 241, 158–71. (10.1016/j.jtbi.2005.11.015) 16386275

[33] Sapareto SA, Hopwood LE, Dewey WC, Raju MR, Gray JW 1978 Effects of hyperthermia on survival and progression of chinese hamster ovary cells. Cancer Res. 38, 393–400. (10.1080/02656730701769841) 563767

[34] Xu M, Myerson RJ, Straube WL, Moros EG, Lee JT, Roti Roti JL 2002 Radiosensitization of heat resistant human tumour cells by 1 h at 41.1°C and its effect on DNA repair. Int. J. Hyperthermia Hyperthermia 18, 385–403. (10.1080/0265673021014690) 12227926

[35] Myerson RJ, Roti JLR, Moros EG, Straube WL, Xu M 2004 Modelling heat-induced radiosensitization: clinical implications. Int. J. Hyperthermia 20, 201–212. (10.1080/02656730310001609353) 15195514

[36] van Leeuwen CM, Crezee J, Oei AL, Franken NAP, Stalpers LJA, Bel A, Kok HP 2017 3D radiobiological evaluation of combined radiotherapy and hyperthermia treatments. Int. J. Hyperthermia 33, 160–169. (10.1080/02656736.2016.1241431) 27744728

[37] Mouratidis PXE, Rivens I, ter Haar G 2015 A study of thermal dose-induced autophagy, apoptosis and necroptosis in colon cancer cells. Int. J. Hyperthermia 31, 476–88. (10.3109/02656736.2015.1029995) 25974074

[38] van Leeuwen CM, Oei A, ten Cate R, Franken NAP, Bel A, Stalpers LJA, Crezee J, Kok HP 2017 Measurement and analysis of the impact of time-interval, temperature and radiation dose on tumour cell survival and its application in thermoradiotherapy plan evaluation. Int. J. Hyperthermia 1–9. (10.1080/02656736.2017.1320812) 28540813

[39] ter Haar G, Coussios C 2007 High intensity focused ultrasound: past, present and future. Int. J. Hyperthermia 23, 85–87. (10.1080/02656730601185924) 17578334

[40] ter Haar G, Coussios C 2007 High intensity focused ultrasound: physical principles and devices. Int. J. Hyperthermia 23, 89–104. (10.1080/02656730601186138) 17578335

