# Supplementary material for: Combining radiation with hyperthermia: a multiscale model informed by in vitro experiments
Source: J R Soc Interface. 2018 Jan 17;15(138):20170681. doi: 10.1098/rsif.2017.0681 (PMC5805969; doi:10.1098/rsif.2017.0681)
Supplement: Sensitivity analysis of the parameters used [file rsif20170681supp1.pdf]

Appendix A: Sensitivity analysis of the parameters used

The choice of the fixed variables $p_{senescence}$, and a constant ratio of 1.5 for the radio-sensitivity in different cell cycle stages was first verified by a sensitivity analysis using a one-at-a-time approach showing the effect of these parameters on the simulation result (see figure 1). For $p_{senescence}$, values in the range between 0 and 20% were tested since we assumed a relatively small contribution of senescent cells to the overall cell population. Relative ratios of sensitivities at different cell cycle stages ranged from 1 to 10. Neither parameter greatly influenced the simulation results for short term, single fraction experiments, but may be of importance for long-term simulations, or in the simulation of fractionated treatments.

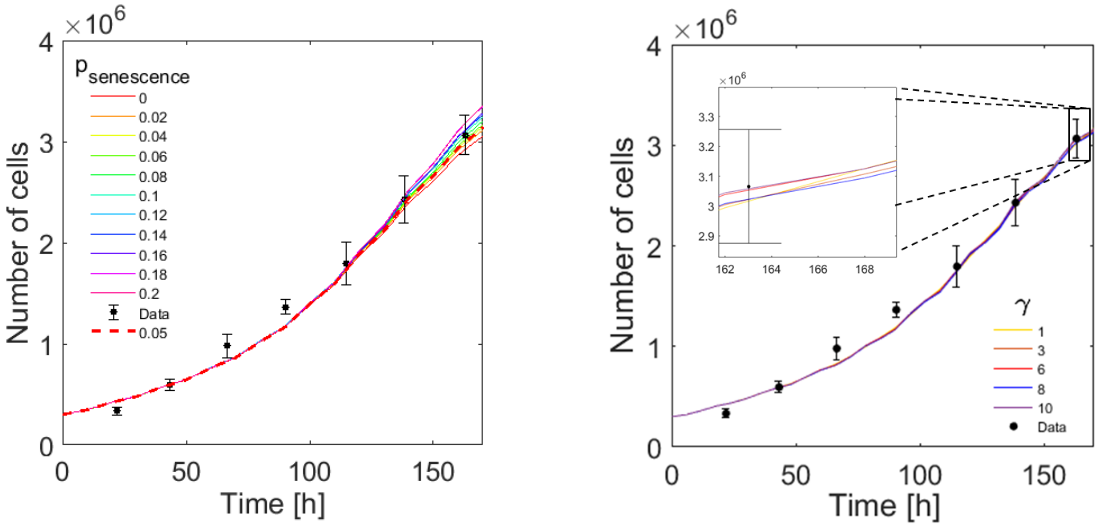

(a) (b)

Figure 1. Sensitivity analysis of the parameters $p_{senescence}$ ranging from 0 to 20% (a), and the relative ratio of radiosensitivity (γ) at different cell cycle stages in the range of 1 to 10 (b). Calibrated simulation results of a growth curve of $2.6 \cdot 10^5$ irradiated (5 Gy) HCT116 cells seeded in 6-well plates with a diameter of 34.8 mm (34.8 mm correspond to a grid diameter of 2900 voxels) are shown. Changes in the probability of senescent rather than giant cells only influences the plateau level of the growth curve. Differences in radiosensitivity between different cell cycle stages result in a more or less pronounced synchronization of the cell cycle of all cells, expressed as a low level oscillation of the growth curve. Since these variations in cell number are less than the range of uncertainties in the cell counts, the assumption of a constant ratio is justifiable.

Furthermore, a variance-based sensitivity analysis according to the methods described in [1] was performed for RT (5 Gy), HT (5 min at 46°C) and RTHT (2 Gy and 5 min at 46°C) treatments. Here, depending on the treatment used, four to eleven parameters were included to assess the influence of their uncertainty on the uncertainty of the output measure. The

sum over all reference data time points of the normalized, squared difference of the number of cells simulated using mean parameter values, N_{mean}, and actual number of cell obtained for each run, N, was used as an output measure.

$$O = \sum \frac{(N_{mean} - N)^2}{N_{mean}^2} \tag{A1}$$

Table I lists the respective parameters included in the simulation for each treatment, together with the uncertainty intervals used and resulting first order and total sensitivity coefficients. It was found that the initial number of cells, as well as the α parameters for both irradiation and heat treatments were most influential.

Table I. Parameters, total (S_T), and first order (S_1) sensitivity indices for three treatment modalities: 5 Gy irradiation (RT), heating for 5 minutes at 46°C (HT), and combination treatment of 2 Gy irradiation and heating for 5 minutes at 46°C (RTHT).

Parameter	RT			HT			RTHT		
	value(range)	S_T	S_1	value(range)	S_1	S_T	value(range)	S_1	S_T
$\alpha_{RT}[Gy^{-1}]$	0.5(0.41,0.59)	0.29	0.04	-	-	-	0.5(0.41,0.59)	0.04	0.02
$\beta_{RT}[Gy^{-2}]$	0.042(0.034,0.051)	0.08	0.02	-	-	-	0.042(0.034,0.051)	0.03	0.01
γ_{RT}	1.5(1,5)	0	0	-	-	-	1.5(1,5)	0.02	0.02
$p_{mitoticCat}$	0.2(0.18,0.22)	0.37	0.02	-	-	-	0.2(0.18,0.22)	0.04	0.01
$p_{senesence}$	0.05(0,0.2)	0.04	0.01	-	-	-	0.05(0,0.2)	0.03	0.01
k_{delay}	$9(8.1,9.9)\cdot10^{-3}$	0.36	0.03	-	-	-	$9(8.1,9.9)\cdot10^{-3}$	0.05	0.01
$\alpha_{0,HT}[min^{-1}]$	0.05(0.03,0.07)	-	-	-	0.95	0.62	0.05(0.03,0.07)	0.96	0.7
$\alpha_{0,HT}^2/\beta_{HT}$	-	-	-	1.95(1.5,2.5)	0.08	0	1.95(1.5,2.5)	0.15	0.03
γ_{HT}	-	-	-	1.5(1,5)	0.22	0.04	1.5(1,5)	0.03	0.01
N_0	$3(2.7,3.3)\cdot10^5$	0.6	0.15	$3.5(3.15,3.85)\cdot10^5$	0.07	0	$2.6(2.34,2.86)\cdot10^5$	0.11	0.03
a	-	-	-	-	-	-	0.015(0.013,0.017)	0.04	0.01

[1] Satelli A, Annono P, Azzinin I, Campolongo F, Ratto M, Tarantola S. 2010 Varianance based sensitivity analysis of model output. design and estimator for the total sensitivity index. *Computer Physics Communications* **181**, 259–270, doi:10.1016/j.cpc.2009.09.018.
